# A membrane localized RTX-like protein mediates physiochemical properties of the *Pantoea stewartii* subsp. *stewartii* cell envelope that impact surface adhesion, cell surface hydrophobicity and plant colonization

**DOI:** 10.1186/s12866-024-03516-w

**Published:** 2024-09-28

**Authors:** Polrit Viravathana, Lindsey P. Burbank, Barbara Jablonska, Qiang Sun, M. Caroline Roper

**Affiliations:** 1 Department of Microbiology and Plant Pathology, University of California, Riverside, CA 92521 USA; 2 USDA Agricultural Research Service, San Joaquin Valley Agricultural Sciences Center, Parlier, CA 93648 USA; 3 Department of Biology, University of Wisconsin, Stevens Point, WI 54481 USA

## Abstract

*Pantoea stewartii* subsp. *stewartii* (*Pnss*), is the bacterial causal agent of Stewart’s wilt of sweet corn. Disease symptoms include systemic wilting and foliar, water-soaked lesions. A Repeat-in-toxin (RTX)-like protein, RTX2, causes cell leakage and collapse in the leaf apoplast of susceptible corn varieties and is a primary mediator of water-soaked lesion formation in the *P. stewartii*-sweet corn pathosystem. RTX toxins comprise a large family of proteins, which are widely distributed among Gram-negative bacteria. These proteins are generally categorized as cellulolysins, but the Biofilm-Associated Proteins (Bap) subfamily of RTX toxins are implicated in surface adhesion and other biofilm behaviors. The *Pnss* RTX2 is most phylogenetically related to other Bap proteins suggesting that *Pnss* RTX2 plays a dual role in adhesion to host surfaces in addition to mediating the host cell lysis that leads to water-soaked lesion formation. Here we demonstrated that RTX2 localizes to the bacterial cell envelope and influences physiochemical properties of the bacterial cell envelope that impact bacterial cell length, cell envelope integrity and overall cellular hydrophobicity. Interestingly, the role of RTX2 as an adhesin was only evident in absence of exopolysaccharide (EPS) production suggesting that RTX2 plays a role as an adhesin early in biofilm development before EPS production is fully induced. However, deletion of *rtx2* severely impacted *Pnss’* colonization of the xylem suggesting that the dual role of RTX2 as a cytolysin and adhesin is a mechanism that links the apoplastic water-soaked lesion phase of infection to the wilting phase of the infection in the xylem.

**Supplementary Information:**

The online version contains supplementary material available at 10.1186/s12866-024-03516-w.

## Background

RTX proteins comprise a large family of proteins that are widely distributed among Gram-negative bacteria and have diverse functional roles. The RTX protein family is divided into a number of subfamilies that includes pore-forming toxins like hemolysins and leukolysins, as well as proteases, lipases, adenylate cyclases, adhesins and cell surface proteins [1, 2]. One subfamily of repetitive RTX adhesins, collectively called *b*iofilm *a*ssociated *p*roteins (Bap) function as loosely attached adhesins and play a role in biofilm development in several bacterial systems such as *Pseudomonas fluorescens*, *Escherichia coli*, *Acinetobacter baumanii*, and *Staphylococcus aureus* [3–5]. Generally, RTX proteins contain glycine-aspartate (GD)-rich peptide repeats with the consensus sequence G-G-X-G-(N/D)-D-X-(L/I/F)-X (X denotes any amino acid) that play a role in binding of calcium ions to facilitate proper protein folding and are often secreted via the Type I secretion system [1, 6–9]. Most RTX toxins have been characterized in mammalian bacterial pathogens, but several plant pathogenic bacteria, such as *Pantoea stewartii* subsp. stewartii (*Pnss*), *Pectobacterium atrosepticum*, *Xanthomonas oryzae* pv. *oryzae*, *Ralstonia solanacearum* and *Xylella fastidiosa* also encode RTX toxins, but many of these are uncharacterized [1, 10–14]. Interestingly, all of these bacteria have associations with the xylem suggesting that RTX toxins facilitate plant xylem colonization.

*Pnss* is a Gram-negative bacterial phytopathogen that causes Stewart’s wilt of sweet corn. The bacteria are introduced into the plant primarily through the feeding wounds created by its insect vector, the corn flea beetle (*Chaetocnema pulicaria*) [15, 16]. *In planta*, *Pnss* colonizes both the leaf apoplast and the xylem of corn plants, causing leaf blight and wilt symptoms, respectively. During the leaf blight phase, the bacteria cause cellular damage that manifests as water-soaked lesions in young plants. The bacteria then preferentially colonize the xylem where they move systemically throughout the plant and form robust biofilms encased in copious amounts of stewartan exopolysaccharide (EPS). It is thought that these dense, mucoid biofilms block the xylem and impede water flow that leads to characteristic wilting that occurs during Stewart’s wilt [15, 17, 18]. We previously demonstrated that a Repeat-in-toxin (RTX)-like protein, designated RTX2, facilitated water-soaked lesion formation and apoplastic colonization in susceptible corn seedlings [13] in concert with the Type III secreted effector, WtsE, that is linked to water-soaking [30]. Mechanistically, we proposed a model where RTX2 serves as the primary cell lysis factor during water-soaked lesion formation where it contributes to cellular collapse and electrolyte leakage in the apoplast [13].

The *Pnss* RTX2 protein also has large repetitive adhesin motifs homologous to hemagglutinins and to the Bap subfamily of RTX proteins leading us to hypothesize that it plays a pleiotropic role as an adhesin and a cytolysin in the *Pnss* pathosystem. Its orthologs include the large repetitive protein YeeJ from *P. ananatis*, and a putative hemagglutinin hemolysin adhesin–related protein from *Erwinia billingiae*. The *Pnss* RTX2 toxin contains five putative Ca^2+^ binding domains, a polycystic kidney disease (PKD) domain, an autotransport domain and C-terminal transmembrane domains [13]. The autotransport domain suggests RTX2 is autotransported out of the cell rather than secreted by the Type I secretion system like prototypical RTX proteins. The PKD domain is predicted to mediate interactions with other proteins and with carbohydrates [13, 19]. Five C-terminal transmembrane domains suggests it localizes to the cell envelope. In this study we determined that RTX2 localized to the cell envelope of *Pnss* where it contributes to overall cellular charge, cell surface hydrophobicity and cell length. Moreover, RTX2 had a nuanced role as a surface adhesin in vitro but deletion of *rtx2* had severe consequences in xylem colonization when directly inoculated into the xylem. These data support our hypothesis that RTX2 plays a pleiotropic role during the infection process where it facilitates water-soaked lesion formation in the apoplastic environment and systemic biofilm colonization in the xylem that leads to wilting.

## Methods

### Bacterial strains, growth conditions, and strain construction

All *Pnss* strains were grown on nutrient agar (Difco Laboratories, Detroit) at 28 °C and *E. coli* strains were grown on LB at 37 °C. Luria-Bertani (LB) broth was supplemented with 0.2% glucose (final concentration) where indicated. All pertinent strains of *Pnss* and *E. coli* are listed in Table 1. When needed and appropriate, the following antibiotics were supplied in the media: nalidixic acid, 30 µg/ml ampicillin, 100 µg/ml, kanamycin, 30 µg/ml, and tetracycline, 30 µg/ml (all final concentration). The *E. coli* S17-1λ strain served as a donor for conjugal transfer. The *Δrtx2/Δwceo* mutant was constructed by introducing the *Δrtx2* deletion construct from Roper et al., 2015 [13] into the *Δwceo* mutant from Carlier et al., 2009 [26].

**Table 1:** Bacterial strains and plasmids

Strain or Plasmid	Relevant Genotypes	Source
*Pantoea stewartii* subsp. *stewartii*		
DC283	Wild type, Nal^R^	[41]
*Δrtx2* (CR54)	Nal^R^, knockout in *rtx2*	[13]
*Δwceo*	Nal^R^, knockout in *wceo*	[26]
*Δrtx2/ Δwceo* (CR59)	Nal^R^, knockout in *wceo / rtx2*	This study
*Δrtx2/rtx2*^*+*^ (CR64)	*Δrtx2*, pMCR29, parental strain DC283	[13]
DC283 w/ pBBR1-MCS4	Nal^R^, Amp^R^	This study
*Δrtx2 w/* pBBR1-MCS4	*Δrtx2*, Nal^R^, Amp^R^	This study
*Δwceo w/* pBBR1-MCS4	*Δwceo*, Nal^R^, Amp^R^	This study
*Δrtx2/ Δwceo w/* pBBR1-MCS4	*Δrtx2/ Δwceo*, Nal^R^, Amp^R^, Kan^R^	This study
*Δrtx2/ Δwceo/ rtx2* ^*+*^	*Δrtx2/ Δwceo*, Nal^R^, Amp^R^, Kan^R^	This study
	pMCR29, parental strain DC283	
*Escherichia coli*		
S17-1λ	RP4, Mob+, Sm^R^	[42]
*Plasmids*		
pHC60	Broad host range vector carrying *gfp, tet*^*R*^	[43]
pMCR29	*rtx2* cloned into pBBR1	[13]
pBBR1-MCS4	Broad host range vector, Amp^R^	[44]

### Qualitative and quantitative assessments of cell size

Individual strains were inoculated into LB broth supplemented with 0.2% glucose (LBG) and grown overnight at 28 °C at 180 rpm. Cultures were sub-cultured 1:20 into fresh media of the same type and propagated to mid-log phase. Cells were harvested by centrifugation at 2,150 x g for 5 min, and washed with sterile 1X phosphate buffered saline (PBS), pH 7.4. Cells were resuspended in sterile 1X PBS, pH 7.4 and adjusted to OD_600nm_ = 0.3. Individual cell suspensions were then inoculated onto separate acid washed glass-slides covered with sterile 0.1 mg/ml poly-L-lysine (Cultrex, R&D Systems, Minneapolis, MN, Catalog #3438-100-01) and allowed to sit for 30 min at 28 °C. Samples then underwent critical point drying with a Tousimis 815 Critical Point Dryer (Tousimis Research Corp., Rockville, MD) and sputter coated with platinum and palladium, then viewed with a Thermo Fisher Scientific NNS450 (Thermo Fisher Scientific, Inc., Waltham, MA) scanning electron microscope (SEM) at the University of California, Riverside Central Facility for Advanced Microscopy and Microanalysis.

For quantitative assessments of cell length via confocal microscopy: Wild type and *Δrtx2* strains containing a plasmid constitutively expressing GFP (pHC60) were grown overnight in LBG and 30 µg/ml tetracycline at 28 °C with shaking at 180 rpm. Cultures were then diluted 1:20 in the same media type and allowed to grow at the same condition until mid-log phase was reached. One milliliter of each strain was individually spun down in a microcentrifuge, washed once and then resuspended in 150 µl of sterile 1X PBS, pH 7.4. Two µl of each suspension was inoculated onto individual 2% agarose pads and imaged with a confocal inverted Zeiss 880 Airyscan UV PALM. Using a 40X water immersion lens, 3 randomly selected fields were taken for analysis with Imaris x64^®^ software (version 9.1.2; Bitplane, Zurich, Switzerland). From each field, ten cells were randomly selected and the length was measured. The experiment was repeated 3 times (*n* = 30 for each strain). Statistical significance was determined via t-test (GraphPad Prism v.10).

Particle size of cell suspensions was quantified using a ZetaPal zeta potential analyzer (Brookhaven Instruments Corporation, Holtsville, NY). Single colonies of either wild type or the *Δrtx2* mutant were grown in LBG overnight at 28 °C with shaking at 180 rpm. Cultures were sub-cultured into fresh LBG at a final dilution of 1:20 and allowed to grow at the same conditions until mid-log phase was reached. Cells were then harvested by centrifugation at 2,150 x g for 10 min. Cell pellets were washed and then resuspended to OD_600nm_ = 0.3 in sterile 10 mM KCl, pH 5.28. Results were based on 3 biological replicates and the experiment was repeated 3 times (*n* = 9). Statistical significance was determined via t-test (GraphPad Prism, version 10).

### Cellular localization of RTX2

The complemented *Δrtx2* mutant strain (*Δrtx2/rtx2*^*+*^) and *Δrtx2* (with empty low copy number pBBR1-MCS4 vector) were propagated at 28 °C for 3 days on nutrient agar supplemented with nalidixic acid, 30 µg/ml. Cells were then harvested with sterile 1X PBS, pH 7.4. Cells were pelleted by centrifugation at 3,836 x g for 10 min and stored at -80 °C. Cells were then thawed on ice and resuspended at a concentration of 0.5 g/ml with Expedeon Proteoloc™ EDTA-free Proteinase Inhibitor Cocktail (Abcam, Inc., Waltham, MA). Following lysozyme treatment for 30 min at 4 °C, the cellular suspension was lysed using a french press. Cell envelope fractions were prepared as per Bennion et al., 2010 [35] with the following modifications: membrane and cytosolic fractions were separated by ultracentrifugation at 105,000 x g at 4 °C for 60 min. The membrane (pellet) fraction was resuspended in tris-buffered saline, pH 7.5. Protein in the cytosolic (supernatant) fraction was precipitated by trichloroacetic acid precipitation. In brief, 100% (weight/volume) of trichloroacetic acid was added to the sample to a concentration of 20%, then incubated on ice for an hour. After centrifugation at 27,000 x g for 10 min, the resulting protein pellet was washed 3 times with ice cold 0.01 M HCl/90% acetone, then air dried [45]. For immunoblotting, the above samples were mixed with 2X Laemmli loading dye (Bio-Rad, Inc., Hercules, CA, Catalog # 1610737), and boiled for 5 min. Samples were loaded onto a 4% stacking and 6% resolving SDS-PAGE gel and transferred onto a 0.2 μm polyvinylidene fluoride membrane and blocked with 5% dried milk in tris-buffered saline, pH 7.4 with 0.1% Tween 20 (TBST). A rabbit polyclonal antibody raised against a synthetic peptide derived from RTX2 (SAELAFTVDNTGSSVALSPVG; both synthesized by Genscript, Piscataway, NJ) in TBST and 5% milk was used as the primary antibody (500 ng/ml, final concentration). Goat anti-rabbit IgG conjugated with horseradish peroxidase (Agrisera, Vannas, Sweden, Cat# AS09 602) was used as the secondary antibody. The blot was developed with a Pierce™ ECL Western Blotting Substrate (Thermo Fisher Scientific, Inc., Waltham, MA) and read with a Bio-Rad ChemiDoc XRS+ (Bio-Rad, Inc., Hercules, CA).

### Bacterial adhesion to hydrocarbon assay

Single colonies of either wild type or *Δrtx2* were grown in LBG broth overnight at 28 °C while shaking at 180 rpm. Cultures were sub-cultured into fresh LBG broth at a final dilution of 1:20 and grown to mid-log phase. Cells were then harvested by centrifugation at 2,150 x g for 10 min. Cell pellets were washed and resuspended to OD_600nm_ = 0.3 in phosphate urea magnesium buffer, pH 7.1. Two ml of cell suspension were aliquoted into sterile glass test tubes and the OD_600nm_ was measured using a Thermo Scientific Biomate 3 spectrophotometer (Thermo Fisher Scientific, Waltham, MA). This measurement served as the initial optical density reading (ODI). Following this, 2 ml of either dodecane or n-hexadecane was added to each separate tubes of cell suspension and vortexed for 2 min. After allowing the phases to separate for 2 h, the final optical density reading (ODF) was taken. Tubes with no hydrocarbon added served as negative controls. The percentage of cell surface hydrophobicity of each strain was calculated as follows: [(ODI-ODF)/ODI] x 100 of treatment cells - [(ODI-ODF)/ODI] x 100 of control cells [28, 36]. Results are 3 replicates per strain and the experiment was repeated 4 times (*n* = 12 per treatment). Data were analyzed using a Mann-Whitney test (GraphPad Prism, v.10).

### Polymyxin B sensitivity

Single colonies of wild type, and *Δrtx2* were propagated overnight in LBG broth. The following day, cultures were diluted 1:20 and inoculated into fresh LBG broth in 96-well U-bottom microplates (Falcon, # 353077) to a final volume of 150 µl. Cells were treated with final concentrations of Polymyxin B (0 µg/ml (Negative Control), 6.25 µg/ml, 12.5 µg/ml, and 25 µg/ml). Growth was assessed in a Tecan Infinite F200 microplate reader (Tecan SP, Inc., Baldwin Park, CA) using an absorbance reading of OD_595nm_ at room temperature. Measurements were taken every hour for 24 h, with 30 s of orbital shaking with an amplitude of 6 mm prior to each reading. Growth curves were based on 3 biological replicates and the experiment was repeated 3 times (*n* = 9 per treatment).

To evaluate cell survival following polymyxin B treatment, *Δrtx2* and wild type cells that were challenged with polymyxin B as described above were serially diluted with 1X phosphate buffered saline (PBS, pH 7.4), plated onto nutrient agar with 30 µg/ml, nalidixic acid (*Pnss* DC283 is Nal^R^), and incubated at 28 °C for 2 days. Percent survival was determined by dividing viable cell counts of polymyxin B challenged bacteria by mock (negative control) viable cell counts. Results are based on 3 replicates for each strain and the experiment was repeated 3 times (*n* = 9 per treatment). Plate count results analyzed via t-test (GraphPad Prism v.10).

### Surface adhesion assays

Adhesion assays were conducted on acetone-etched polystyrene plates to enhance surface attachment. Polystyrene plates (Greiner Bio-One North America, Inc., Monroe, NC, #655101) were treated with acetone for 10 s, inverted to allow evaporation of residual acetone and UV-sterilized for 1 h [46]. Single colonies of wild type, *Δrtx2*, *Δwceo*, *Δwceo/Δrtx2* all carrying empty vector plasmid pBBR1-MCS4, as well as *Δrtx2/rtx2*^+^, and *Δwceo/Δrtx2/ rtx2*^+^ were propagated overnight at 28 °C in LBG broth supplemented with 100 µg/mL ampicillin while shaking at 180 rpm. Overnight cultures were pelleted by centrifugation (2,150 x g for 10 min) and washed twice with sterile 1X PBS, pH 7.4 adjusted to OD_600nm_ = 0.5 and diluted 1:10 in fresh LBG broth supplemented with 100 µg/mL ampicillin. 150 µl of this inoculum was placed in the acetone treated microplate and incubated statically at 28 °C for 48 h. Absorbance readings were measured at OD_595nm_ in a Tecan Infinite M Plex microplate reader (Tecan SP, Inc., Baldwin Park, CA). Following this, planktonic cells and culture medium were removed and 200 µl of a filtered 1% crystal violet ethanol solution was added to each well and incubated statically for an hour. The crystal violet solution was removed and each well was then washed three times with 200 µl sterile water with agitation for 30 s and dried overnight. The crystal violet was then solubilized by adding 200 µl of a 30% acetic acid solution per well and shaken at 100 rpm for 1 h. The acetic acid solution from each well was further diluted 1:10 in 30% acetic acid in an untreated, polystyrene, 96-well microplate, mixed, and the absorbance reading were taken at OD_595nm_. The adhesion value was calculated as follows: (OD_595_CV-media value)/ (OD_595_Cell growth-media value) [37]. Results are based on 36 replicates per strain and the entire experiment was repeated 5 times (*n* = 180 per strain). Statistical analysis was performed using a linear mixed effects model, where adhesion value was the response variable, strain, biological replicate, and their nested interactions were fixed effects, and plate number and the interactions between plate and strain were random effects. Post hoc analysis was performed using the least-squares means method and was corrected for multiple comparisons using the sidak method. Statistical analysis was performed with R version 4.0.5 with Ime4 and emmeans packages.

### Confocal microscopy of in vitro biofilms

Assays were based on Koutsoudis et al., 2006 with modifications [25]. In brief, single colonies of *Δwceo*,* Δwceo/Δrtx2*, wild type and *Δrtx2*, all carrying plasmid pHC60, were grown separately overnight, at 28 °C in LBG with 30 µg/mL tetracycline shaking at 180 rpm. After resuspending (by gentle inversion until solution was homogenous, without using a vortex), centrifugation (2,150 x g for 10 min) and washing twice in sterile 1 X PBS, pH7.4, these overnight cultures were all adjusted to OD_600nm_ = 0.5, then diluted 1:10 in fresh LBG with 30 µg/mL tetracycline. Following this, 7.5 ml of cell suspension was placed in a sterile 50 ml conical tube along with an autoclaved, confocal grade coverslip (Electron Microscopy Sciences, Hatfield, PA, Cat # 72204-01). Prior to use, coverslips were gently etched with P220 grade sandpaper, rinsed with sterile water and sterilized by autoclave. Tubes were then incubated statically for 96 h at 28 °C. After gently rinsing the inoculated coverslip with sterile water, the biofilm formed at the location of the liquid air interface was analyzed by placing the incubated coverslip on a glass slide covered with an adhesive microscope slide grid (Diversified Biotech, Inc., VWR, Visalia, CA, Catalog # 89032-163), and viewed with a Zeiss 880 upright confocal microscope at 20X magnification. A total of 5 biological replicates were imaged with 11 images taken at individual grid points along the length of the liquid-air interface (*n* = 55 measurements per strain). BiofilmQ software developed by Hartmann et al., 2021 [38] was used for quantitative three-dimensional image analysis of biofilms formed in-vitro. The Z-stack images analyses were based on Castro et al., 2023 [39] and formed biofilms were denoised by convolution using the default parameters, while floating cells were removed from images and a threshold of 100 vox was used to remove small artifacts due to noise. Top-hat filter was set to a value of 15 to remove background fluorescence. Images were segmented automatically with a sensitivity of 1.75, and use of the Otsu algorithm [43].

### Scanning electron micrographs of in vivo biofilm formation

Infection of 10-day old *Zea mays* var. Jubilee corn seedlings was performed as per Roper et al., 2015 [13]. At 5 days post inoculation, the entire corn plant was fixed in FAA (formalin-acetic acid-alcohol) for at least 48 h. Three-five 1 mm wide leaf strips were cut from the base part, middle portion, and tip portion of each leaf blade, respectively. The leaf strips were dehydrated through an ethanol series of 70%, 80%, 90%, 95%, 100% and 100% with 15 min at each step. Dehydrated leaf specimens were critical-point dried with Tousimis Autosamdsri-931 (Tousimis Research Corp., Rockville, MD) and then sputter-coated with Au-Pd in a Safematic CCU-010 compact coating unit (Safematic GmbH, Zizers, Switzerland). Coated specimens were examined for the leaf’s transverse surface and photographed under an SEM (Hitachi S3400-II) at the accelerating voltage of 8 kV with a secondary electron detector.

### RNA isolation and cDNA synthesis

Total RNA was extracted from *Pnss* strain DC283 culture grown on AB minimal medium [40] for 2 days at 28 °C. RNA extraction was done according to the protocol of Quick-RNA MiniPrep (Zymo Research Corp., Irvine, CA). RNA samples were treated with Turbo Dnase I (Thermo Fisher Scientific, Inc.,Waltham, MA), following the manufacturer’s protocol to remove trace genomic DNA. cDNA was synthesized from 2 µg of total RNA using 2 pmole of gene-specific primer and SuperScript™ II Reverse Transcriptase (Thermo Fisher Scientific, Inc., Waltham, MA). One-twentieth volume of each cDNA was used as a template for PCR amplification. The gene-specific primers and PCR conditions were as described below. A no reverse transcriptase control (-RT) was used to test for DNA contamination. As a positive control, the primer pairs were first tested by amplifying the fragment from total *Pnss* genomic DNA. Briefly, *Pnss* strain DC283 was grown on Miller’s Luria-Bertani (LB) medium (Difco) for 2 days at 28 °C, and genomic DNA was isolated using DNeasy blood and tissue kit (Qiagen Sciences, Germantown, MD) following the manufacturer’s protocol.

### Co-transcription analysis of *rtx1/rtx2/rcsD/rcsB*

Co-transcription of *rtx1/rtx2/rcsD/rcsB* was assessed because they are genetically linked together. The RTX1-RTX2 coding sequence (CDS) was amplified from a *Pnss* cDNA sample synthesized with RT-RTX2 primer (5-GAGATCAGACTGGTCAACTC-3’). A 573 bp fragment was amplified from this cDNA using Taq DNA Polymerase with standard Taq buffer, 200 µM dNTP’s mix (New England Biolabs, Corp., Ipswich, MA), and with primers RTX1-F2 (5′- CAGCGATGGTGTCCTTAATA-3′) and RT-RTX2 primer (5’-GAGATCAGACTGGTCAACTC-3’) at 0.2 µM final concentration under the following conditions: denaturation at 95 °C for 30s min, followed by 35 cycles of 95 °C for 15 s, 52 °C for 30 s, and 72 °C for 1 min, followed by 1 cycle of final extension at 72 °C for 15 min. The RTX2-RcsD CDS was amplified from a *Pnss* cDNA sample synthesized with RT-RcsD primer (5’- GGCAGGGTCGATATCATAATCAGGC-3’). A 609 bp fragment was amplified from this cDNA, using Taq DNA Polymerase with standard Taq buffer, 200 µM dNTP’s mix (New England Biolabs, Corp., Ipswich, MA M0273L) and with primers RTX2-F2 (5′- ATTCTTTCTGGTCCGGCGTT-3′) and RT-RcsD (5’-GGCAGGGTCGATATCATAATCAGGC-3’) at 0.2 µM final concentration, under the following conditions: denaturation at 95 °C for 30s min, followed by 30 cycles of 95 °C for 15 s, 54 °C for 30 s, and 72 °C for 1 min, followed by 1 cycle of final extension at 72 °C for 15 min. The RcsD- RcsB CDS was amplified from a *Pnss* cDNA sample synthesized with RT-RcsB primer (5’-CGACAGATCAGGATAATGGCGTTTG-3’). A 274 bp fragment was amplified using Taq DNA Polymerase with standard Taq buffer, 200 µM dNTP’s mix (New England Biolabs, Corp., Ipswich, MA M0273L) (NEB N0447L), and with primers at 0.2 µM final concentration: RcsD-F (5′ TCAACTGCTGCAGCAAGGTAACCAA − 3’) and RT-RcsB (5’ CGACAGATCAGGATAATGGCGTTTG-3’), under the following conditions: denaturation at 95 °C for 30s min, followed by 30 cycles of 95 °C for 15 s, 59 °C for 30 s, and 72 °C for 1 min, followed by 1 cycle of final extension at 72 °C for 15 min. Primers were designed with Geneious Prime software version 2019.0.4 and synthesized by Integrated DNA Technologies (Integrated DNA Technologies, Inc., Coralville, IA).

## Results

### Deletion of *rtx2* decreases cell size

**Fig. 1:**
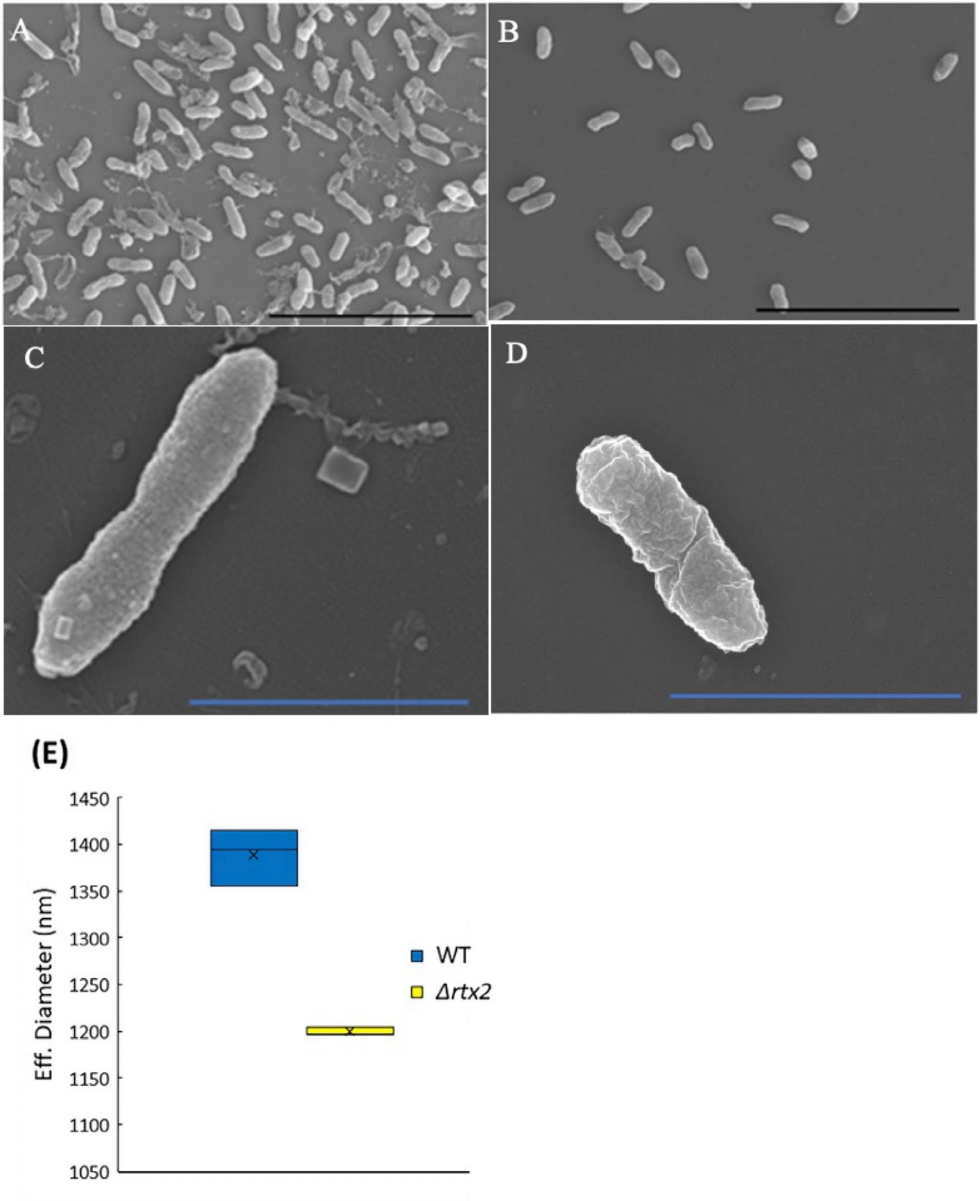
Deletion of *rtx2* resulted in a decrease in cell size. Scanning electron micrographs indicated that the wild type *Pnss* (**A** and **C**, scale bar = 10 μm and 2 μm, respectively) was longer than the *Δrtx2* mutant (**B** and **D**, **E**) Zeta potential measurements indicated that wild type *Pnss* had a significantly larger Eff diameter than *Δrtx2p* = 0.0004 by t-test. Particle size analysis results were based on 3 replicates per strain and the experiment was repeated 3 times (*n* = 9). Significance was determined by t-test. The top and bottom whiskers of the box plot represent the highest and lowest values (excluding outliers), respectively. The average sample value is denoted as an X. The line between the top and bottom of the box represents the median value. The top of the box is the 3rd or upper quartile (25% of observations are greater than this value). The bottom of the box denotes the 1st or lower quartile (25% of observations are lower than this value)

Deletion of *rtx2* caused a decrease in cell length as compared to wild type *Pnss* as indicated by scanning electron micrographs (Fig. 1A-D). Differences in qualitative length were confirmed by confocal microscopy between wild type and the *Δrtx2* strains expressing green fluorescent protein (GFP) (Figure S1). In addition, Zeta Pals particle size analysis indicated that the Δ*rtx2* mutant had a smaller average effective (Eff) diameter when compared to wild type *Pnss* (*p* = 0.0004) (Fig. 1E). Eff diameter is the determined diameter of the bacterial cell in a solution, based on its light scattering properties. Eff diameters can be used as the equivalent diameter of the cell particle and correlates with microscopic measurements [20, 21]. Growth curves for wild type *Pnss* and *Δrtx2* coupled with plate counts every other hour indicated no significant difference in growth rate between the two strains (*data not shown*).

### RTX2 localizes to the cell envelope

**Fig. 2:**
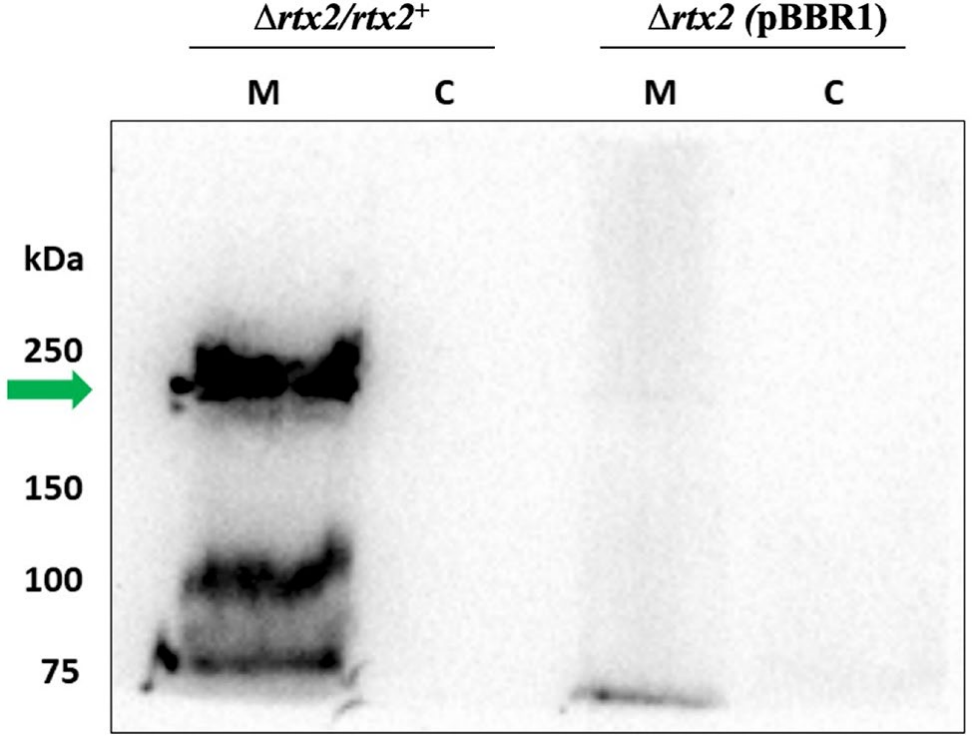
RTX2 localizes to the membrane fraction of the cell. Rabbit polyclonal antibody raised against a peptide of the RTX2 protein (Genscript, Piscataway, NJ) detected a protein of approximately 250 kDa (RTX2 = 249.8 kDa) in the membrane fraction (indicated with a green arrow) of *Pnss (∆rtx2/rtx2*^*+*^). This band was not detected in the cytoplasmic fraction of this strain, or in the membrane or cytoplasmic fractions of the corresponding *Δrtx2* mutant *(∆rtx2* w/ pBBR1 (empty pBBR1-MCS4 vector)). *M* = membrane and *C* = cytoplasmic

Based on the presence of five transmembrane domains, RTX2 was predicted to be localized to the bacterial cell envelope [13]. Western blot analysis using an anti-RTX2 antibody confirmed that RTX2 localizes to the cell envelope in *Δrtx2* complemented with the low copy number pBBR1::*rtx2* plasmid. We were unable to detect RTX2 in the wild type strain of *Pnss* likely because the chromosomal RTX2 is either transiently expressed or expressed in low quantities (*data not shown*). RTX2 was not detected in the cytosolic fraction in any strain including the *Δrtx2* complement carrying the pBBR1::*rtx2* plasmid. RTX2 was not detected in the membrane of the *Δrtx2* mutant carrying the pBBR1-MCS4 empty vector (Fig. 2). The antibody also detected additional bands only in the membrane fraction of the *Δrtx2* complement strain suggesting possible post-translation processing of the RTX2 protein when it associates with the cell envelope.

### Deletion of *rtx2* significantly impacts membrane integrity

**Fig. 3:**
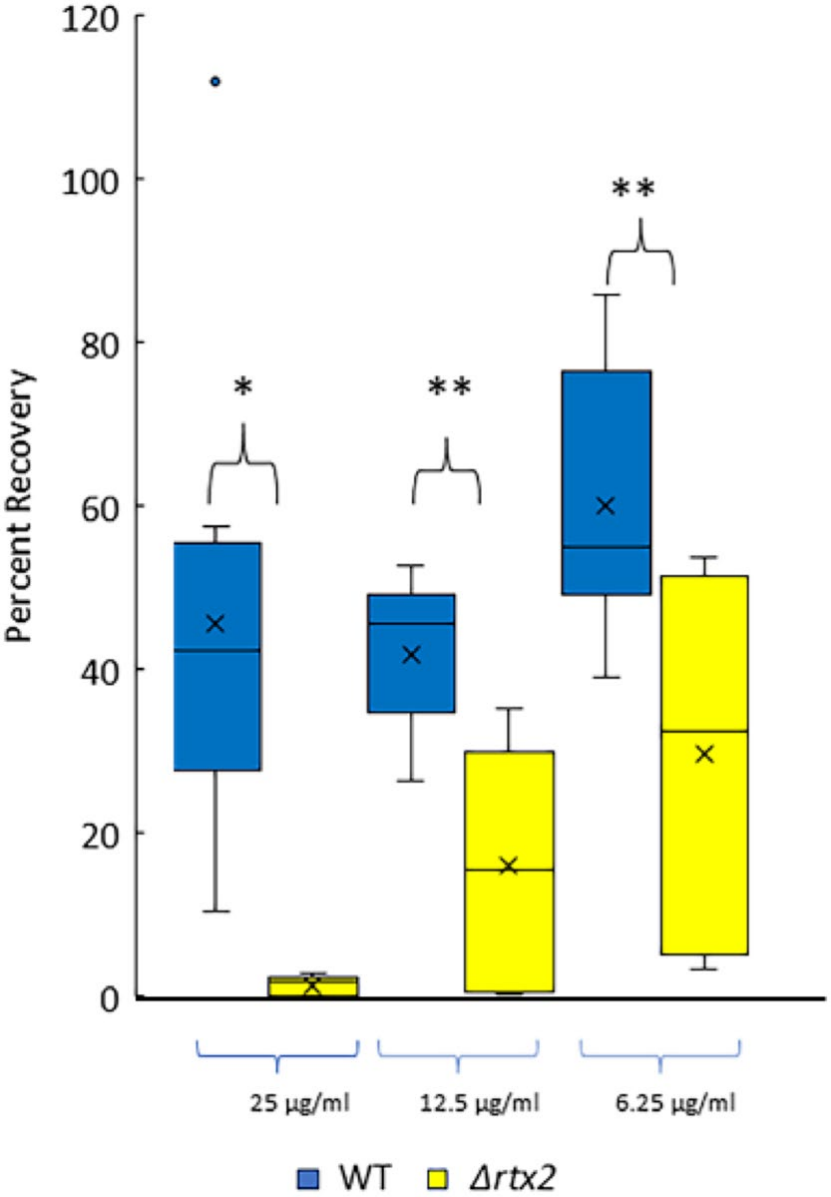
Deletion of *rtx2* increased sensitivity to polymyxin B. End Point plate counts after treatment with increasing concentrations of polymyxin B indicated that the *Δrtx2* mutant is more sensitive to polymyxin B as compared to the wild type * *p* ≤ 0.002, ** *p* ≤ 0.0001 by t-test; *n* = 9 per treatment. Data are from 3 replicates of each strain and the experiment was repeated 3 times. The top and bottom whiskers of the box plot represent the highest and lowest values (excluding outliers), respectively. The average sample value is denoted as an X. The line between the top and bottom of the box represents the median value. The top of the box is the 3rd or upper quartile (25% of observations are greater than this value). The bottom of the box denotes the 1st or lower quartile (25% of observations are lower than this value)

Because RTX2 is a large protein that localizes to the bacterial cell envelope we reasoned that it may contribute to membrane integrity and influences the physiochemical properties of the cell envelope. The antibiotic polymyxin B destabilizes the bacterial outer membrane by binding to the lipopolysaccharide layer and can be used as an indication of membrane integrity [22]. Deletion of *rtx2* resulted in significantly increased sensitivity to polymyxin B at all concentrations tested (25, 12.5 and 6.25 µg/ml) as compared with the wild type parent strain (Fig. 3) indicating that RTX2 is linked to membrane integrity either directly or indirectly.

### RTX2 affects cell surface hydrophobicity

**Fig. 4:**
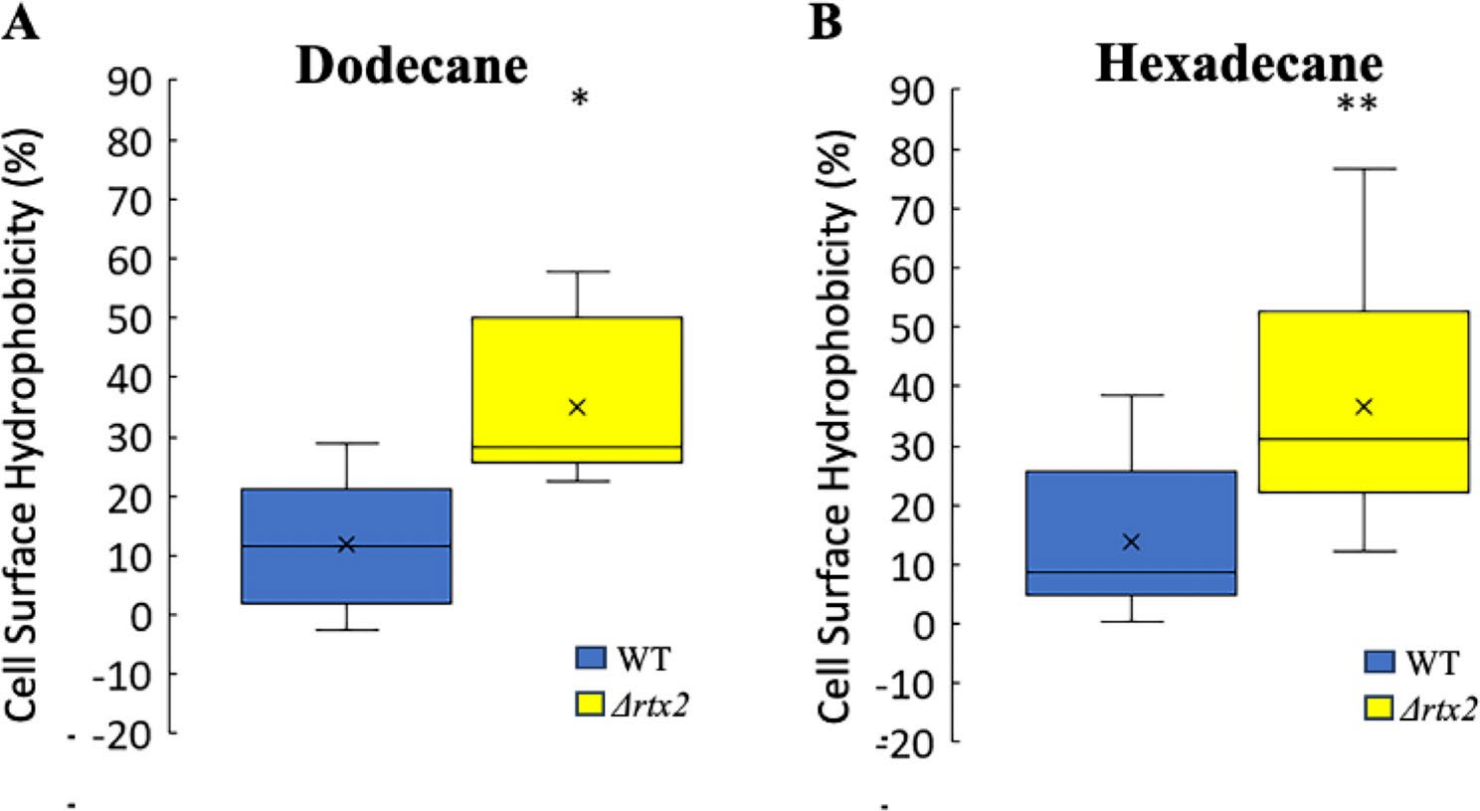
Deletion of *rtx2* increases cell surface hydrophobicity. Bacterial Adhesion to Hydrocarbon (BATH) Assays using the organic solvent **A**) Dodecane or **B**) N-Hexadecane indicated that the *Δrtx2* mutant had increased cell surface hydrophobicity compared to the wild type. * indicates significance at *p* < 0.0001 and ** *p* < 0.002, Mann Whitney; *n* = 12 per treatment. Data are from 3 replicates of each strain and the experiment was repeated 4 times. The top and bottom whiskers of the box plot represent the highest and lowest values (excluding outliers), respectively. The average sample value is denoted as an X. The line between the top and bottom of the box represents the median value. The top of the box is the 3rd or upper quartile (25% of observations are greater than this value). The bottom of the box denotes the 1st or lower quartile (25% of observations are lower than this value)

To determine if RTX2 impacts physiochemical properties of the cell envelope we performed bacterial adherence to hydrocarbons (BATH) assays. This assay quantifies cell surface hydrophobicity by assaying the cell’s affinity to organic hydrocarbons [23, 24]. The more hydrophobic the cell, the more cells will partition to the organic phase, leading to a decrease in optical density in the buffer phase. BATH assays indicated that the *Δrtx2* mutant had higher adherence to the hydrocarbons dodecane and n-hexadecane indicating the cell surface of *Δrtx2* is more hydrophobic than the wild type parental strain *p* < 0.0001 (dodecane) and *p* < 0.002 (hexadecane) (Mann Whitney) (Fig. 4).

### RTX2 is involved in surface adherence in the absence of stewartan exopolysaccharide

**Fig. 5:**
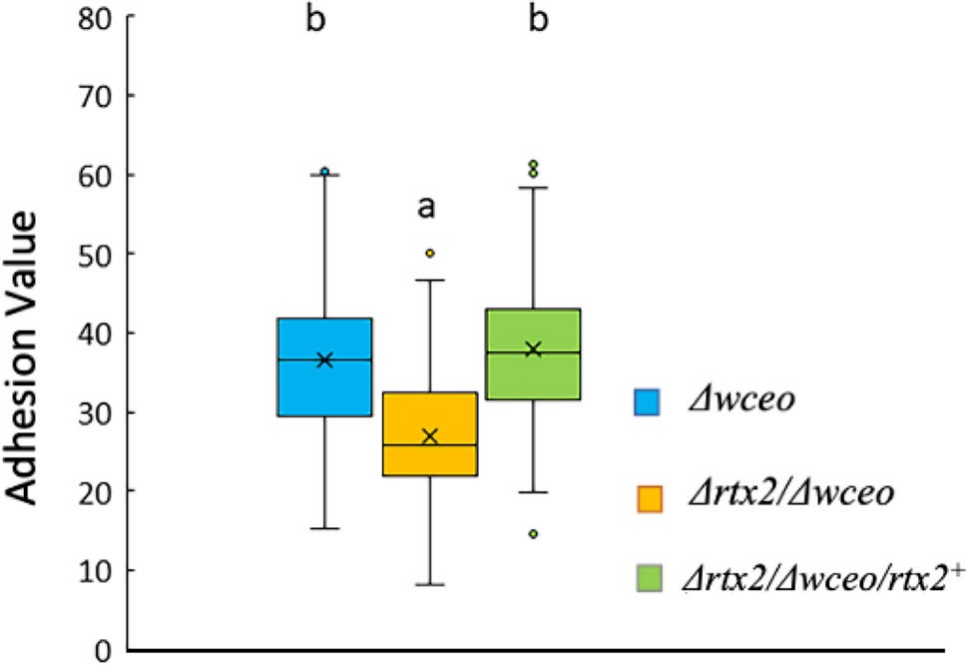
Deletion of *rtx2* decreased in vitro surface adhesion in a non -EPS producing genetic background. In a non-EPS producing (*Δwceo*) genetic background, *Δrtx2/Δwceo* attached to acetone-etched polystyrene significantly less than the parental *Δwceo* strain. Adhesion was restored to parental strain levels in the *Δrtx2/Δwceo/rtx2*^*+*^ strain. Results are based on 36 replicates per strain and the entire experiment was repeated 5 times (*n* = 180 per strain). Statistical analysis was performed using a linear mixed effects model, followed by a post hoc analysis using the least-squares means method with the sidak method to correct for multiple pairwise comparisons. Letters denote significant differences among treatments, means with the same letter are not statistically different. The top and bottom whiskers of the box plot represent the highest and lowest values (excluding outliers), respectively. The average sample value is denoted as an X. The line between the top and bottom of the box represents the median value. The top of the box is the 3rd or upper quartile (25% of observations are greater than this value). The bottom of the box denotes the 1st or lower quartile (25% of observations are lower than this value)

To determine if RTX2 was involved in single cell adhesion to surfaces, we performed a surface adherence assay utilizing acetone-etched polystyrene plates as the surface substratum. This assay quantifies cell-surface adhesion, one of the initial steps in biofilm formation. *Pnss* produces large amounts of fluid stewartan EPS in culture, which can impede surface attachment in vitro and confound crystal violet-based surface adhesion assays [25]. Thus, we opted to test surface adhesion in a *Pnss Δwceo* genetic background that does not produce EPS. The *wceo* gene encodes a glucosyl-transferase required for EPS production in *Pnss* [26]. Deletion of *rtx2* in the *Δwceo* genetic background (*Δrtx2/Δwceo*) resulted in a significant decrease in adhesion compared to the *Δwceo* mutant (Fig. 5). Surface adhesion was restored in the *Δrtx2/Δwceo* strain when *rtx2* was supplied *in trans* on the low copy number pBBR1::*rtx2* (with *rtx2* under the control of a T3 promoter) (Fig. 5).

### RTX2 contributes to 3-dimensional biofilm volume in the absence of stewartan exopolysaccharide

**Fig. 6:**
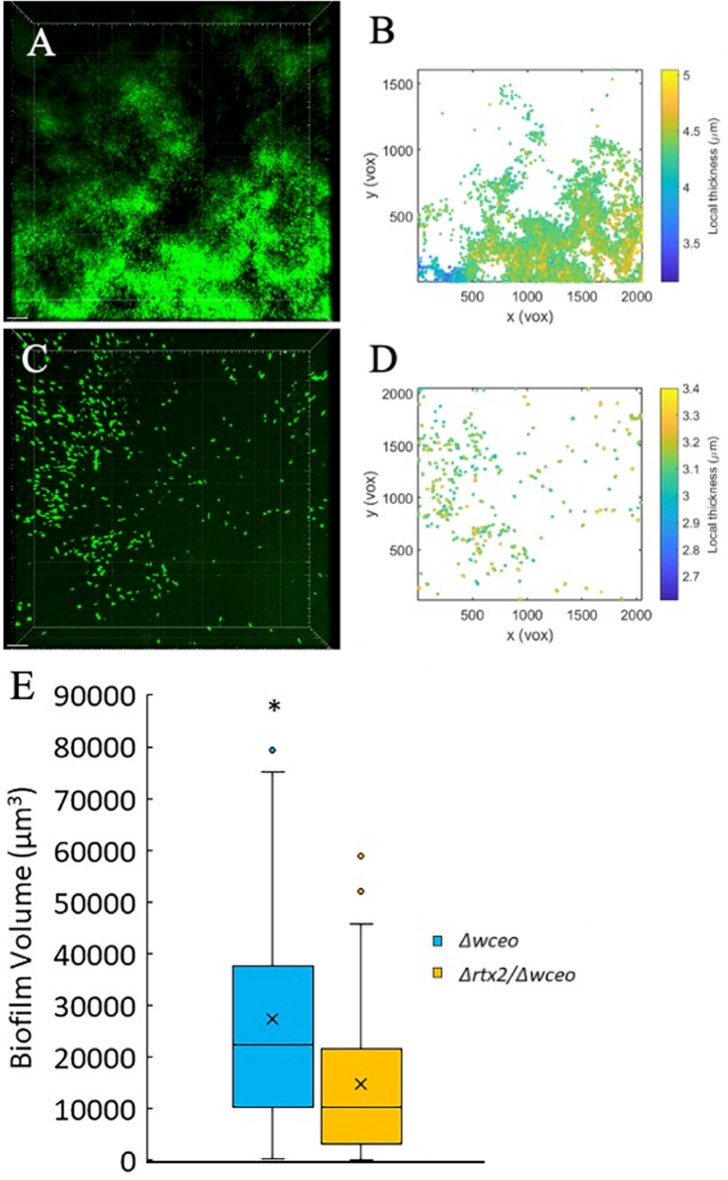
Deletion of *rtx2* reduced in vitro biofilm volume in a non-EPS producing genetic background. Biofilm volume was reduced in vitro when the *Δrtx2* deletion mutation was introduced into a non-EPS producing (*Δwceo*) genetic background. Specifically, the parental strain *Δwceo* (**A** and **B**) had significantly more overall 3-dimensional volume than the **C** and **D**) the *Δrtx2/Δwceo* strain. **E**) Results are based on 11 separate measurements within the biofilm of each strain and the experiment was repeated 5 times. * indicates significance at *p* = 0.0064 via Mann-Whitney Test; *n* = 55. The top and bottom whiskers of the box plot represent the highest and lowest values (excluding outliers), respectively. The average sample value is denoted as an X. The line between the top and bottom of the box represents the median value. The top of the box is the 3rd or upper quartile (25% of observations are greater than this value). The bottom of the box denotes the 1st or lower quartile (25% of observations are lower than this value)

The reduction in 3-dimensional biofilm volume was significant in a non-EPS genetic background. Based on quantification of overall biofilm volume, the *Δrtx2*/*Δwceo* mutant was significantly impaired in building the 3 dimensional architecture of the biofilm when compared to the *Δwceo* strain (Fig. 6). Results were based on measurements within the biofilm and the experiment was repeated 5 times (Mann-Whitney test; *p* = 0.0064; *n* = 55). The wild type strain and *Δrtx2* qualitatively had different biofilm architectures, but quantitatively the biofilm volume between wild type and *Δrtx2* were similar (Figure S2). Results are based on 11 measurements within the biofilm and the experiment was repeated 5 times (Mann-Whitney test *p* = 0.7094; *n* = 55).

### RTX2 is required for xylem colonization

**Fig. 7:**
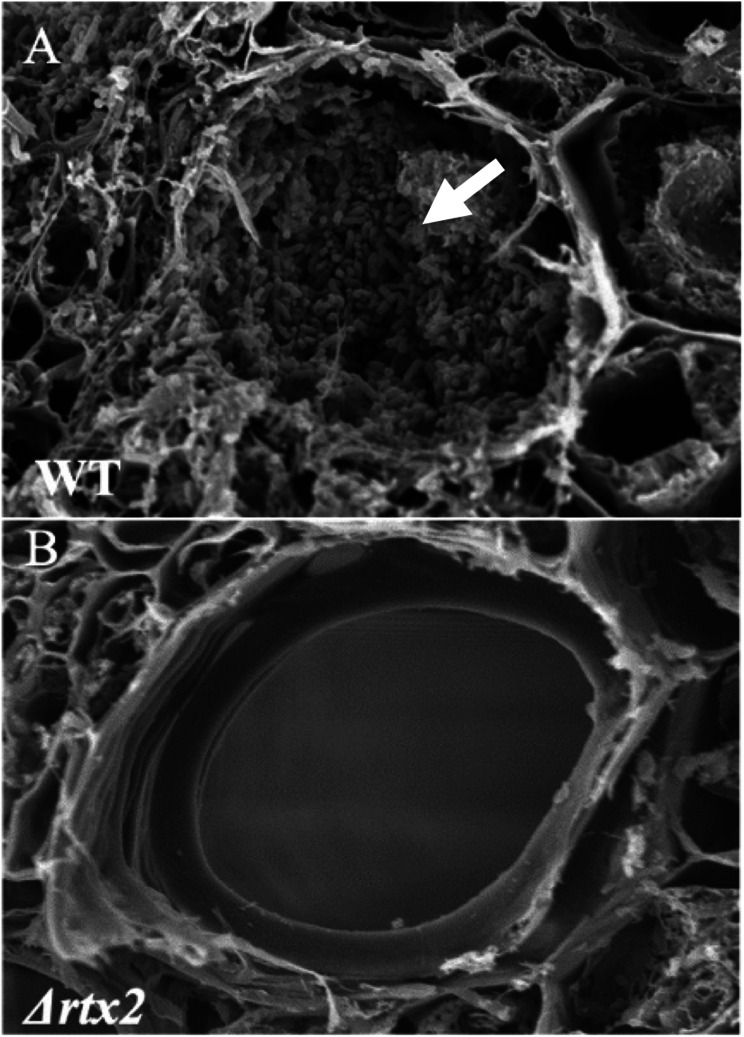
Deletion of *rtx2* abolishes xylem colonization in vivo. Representative SEM Images of **A**) wild type *Pnss* and **B**) *Δrtx2* inoculated plants showed that the RTX2 protein is required by *Pnss* to form the EPS-based biofilm characteristic of Stewart’s wilt. SEM images of inoculated plants, 5 days post-inoculation show that while wild type *Pnss* is found in the xylem (white arrow), the *Δrtx2* mutant is not present in significant numbers to be detected. Scale bar in both images is 5 μm

Depending on the inoculation method used, the apoplastic phase of infection can be separated from the xylem phase of infection. By inoculating the whorls of seedlings, the plants are not wounded and the bacteria only enter the apoplast initially. The scratch-inoculation technique, creates wounds using a syringe needle. The bacterial inoculum is then deposited in the wound, which allows bacteria to colonize both the xylem and the leaf apoplast. This method mimics the natural infection process facilitated by the corn flea beetle feeding behavior and allows assessment of a strain’s ability to cause the wilting that follows WS lesion formation in young seedlings. In our previous work, we demonstrated that the *Δrtx2* mutant did not incite water-soaked lesions or wilt when inoculated using both the whorl or scratch inoculation method despite colonizing the plant tissue to relatively high titers of approximately 2.3 × 10^7^ cfu/g [13]. In this study, we wanted to further explore the *Δrtx2* mutant’s colonization capability of the xylem because of RTX2’s properties as an adhesin. Following needle scratch inoculation, we made cross sections in the middle portion of a leaf blade distal to the inoculation site in 10 day old corn seedlings harvested 5 days post-inoculation. Thus, to be present and observable by SEMs the bacteria needed to move systemically within the xylem. Scanning electron micrographs indicated that wild type *Pnss* formed thick EPS-encased biofilms in the xylem vessel lumen (Fig. 7). Whereas, when the *Δrtx2* was inoculated in the same manner, bacterial cells were not readily observed in the vessel lumen. This indicates that in addition to be compromised on water-soaked lesion formation [13], the *Δrtx2* was also compromised in xylem colonization (Fig. 7).

### *rtx2* is co-transcribed with the *rcsD* and *rcsB* components of the Rcs phosphorelay

Genomic analysis predicted that *rtx2* belongs to a four gene operon that includes the *rcsD* and *rcsB* gene that encode central components of the *R*egulator of *c*apsular *s*ynthesis (Rcs) signal transduction pathway. The Rcs pathway has been well described in *Escherichia coli* as well as *Pnss* where it has tight regulatory control of EPS synthesis as well as other cell surface phenotypes. RcsD is a phosphotransferase and RcsB is a cytoplasmic response regulator [33]. Using a combination of reverse transcription and PCR, we demonstrated that *rtx2* is, indeed, co-transcribed with *rcsD* and *rcsB* (Figure S3).

## Discussion

*In planta*, both the leaf apoplast and the xylem present a mosaic of hydrophilic and hydrophobic surfaces that the bacterium must adhere to during the infection process. Thus, *Pnss’s* ability to change its surface properties during its interaction with different tissue niches in the plant is critical to the infection process. Cell surface hydrophobicity is a multifactorial phenotype dictated by the composition of the bacterial cell surface and is an important parameter that governs bacterial attachment and detachment to biotic surfaces [24]. Hydrophobicity can be impacted by cell envelope structures including lipopolysaccharides and cell surface proteins [44]. Microorganisms can fine tune their cell surface hydrophobicity in response to changes in environmental conditions (temperature, composition of nutrients, etc.) and growth phases that, in turn, affects their adhesion to surfaces [24]. Surface-localized adhesins, such as LapF, from *Pseudomonas putida* and YcfR of *E. coli* contribute to overall cell surface hydrophobicity [27, 28]. The Bap-like protein, Esp, from biofilm forming strains of *Enterococcus faecalis* promotes primary attachment to surfaces and also is an important contributor to cell surface hydrophobicity [29]. Membrane fractionation and immunoblot experiments confirmed that the RTX2 protein localized to the *Pnss* envelope as predicted by the five transmembrane domains in the C-terminus. Moreover, deleting *rtx2* resulted in a significant increase in cell surface hydrophobicity of *Pnss* indicating either that the RTX2 protein imparts hydrophilic properties to the cell surface or that deleting RTX2 exposes hydrophobic entities on the cell surface.

In canonical biofilm development, biofilm initiation is mediated by cell surface adhesins that allow for attachment to surface substrata and cell-cell aggregation that leads to microcolony formation. Once established in a microcolony the biofilm cells differentiate into macrocolonies and, in turn, EPS production is induced that contributes to the building and maintenance of the biofilm architecture [34]. We speculated that RTX2 acts as a primary adhesin that is most relevant during early biofilm establishment. Because of this, we tested the role of RTX2 as an adhesin in absence of stewartan EPS by introducing the *rtx2* deletion mutation into the *Δwceo* genetic background that is defective in EPS production [26]. By knocking out EPS production this physiologically locked the cells in early biofilm establishment which allowed us to characterize RTX2’s role in attachment to surface substrata. Indeed, the *Δrtx2* mutant was similar to the wild type parent when assayed for direct cell-to-surface adhesion properties to polystyrene using a crystal violet assay designed to isolate the initial attachment phase of biofilm formation (*data not shown*). However, the *Δrtx2/Δwceo* strain was significantly impaired in attachment to the polystyrene substrate indicating that RTX2 has adhesive properties that contribute to surface adhesion that were not apparent in the presence of EPS. Moreover, in vitro biofilms formed by *Δrtx2/Δwceo* had significantly less overall volume as compared to *Δwceo* indicating that RTX2’s adhesive properties also have downstream effects on biofilm maturation that were not observed in the wild type genetic background that produces normal levels of EPS.

Colonization of the sweet corn xylem requires adherence to the xylem wall and subsequent spatial and temporal biofilm formation to achieve the systemic colonization that leads to wilting associated with the disease. Interestingly, the *Δrtx2* mutation in a wild type genetic background resulted in a dramatic impairment in xylem colonization indicating that RTX2’s role as an adhesin during early biofilm colonization is crucial for effective biofilm development in vivo in the xylem. Xylem vessels are non-living at maturity and likely present a markedly different surface substratum than the bacteria encounter in the apoplast where they primarily interact with living cells. Based on the inoculation method used, it is possible to separate the apoplastic phase of infection from the xylem phase of infection. Interestingly, when using a needle inoculation method that introduces *Pnss* directly into the xylem and essentially bypasses the apoplast phase of the infection process, the *Δrtx2* mutant strain did not cause wilting symptoms supporting our hypothesis that RTX2 has a pleiotropic role in plant host colonization as a cytolysin and an adhesin that is tissue-type specific. Our data also indicate that deletion of *rtx2* compromises the membrane integrity of the cell making it more susceptible to polymyxin B that targets the cell envelope. We speculate that in addition to its role in adhesion, disruption of membrane integrity in the *Δrtx2* mutant may also make it more susceptible to plant defenses, such as reactive oxygen species, which also impacts its ability to colonize the apoplast and xylem. The role of related Bap proteins in mediating membrane integrity is largely unknown.

Spatiotemporal mathematical models of biofilm assembly predict that cell length has an impact on shaping biofilm structure. Specifically, a longer average cell length yields more rapidly expanding, flatter biofilms than those formed by shorter cells [31]. Deletion of *rtx2* resulted in a significant reduction in bacterial cell size when compared to wild type *Pnss*. We speculate that its role in modulating cell size also is a factor in its role in biofilm formation that is more apparent in vivo than in vitro. It is unknown how this surface-localized protein aids in determination of cell size or why a smaller cell size impacts biofilm development. However, in *Pnss* the *rtx2* gene is co-transcribed in an operon with two components of the *R*egulator of *C*apsular *S*ynthesis (Rcs) phosphorelay regulatory system, the phosphotransferase RcsD, and the response regulator, RcsB [32]. The Rcs phosphorelay is an environmental responsive, multi-component signal transduction system that regulates many cell-surface associated phenotypes that include EPS production, cell division, integrity of the cell envelope, biofilm formation, flagellar genes and virulence factors [32, 33]. Because RTX2 localized to the cell envelope and *rtx2* is co-transcribed with *rcsB* and *rcsD*, we speculate RTX2 may participate in the fine-tuning of the physiochemical properties of the bacterial cell that are controlled by the environmental monitoring Rcs system. Future studies on how RTX2-mediated membrane perturbation may be linked to modulation of the Rcs phosphorelay are warranted.

## Conclusions

RTX2 is a critical pathogenicity factor for *Pnss* and was previously described as a cytolytic toxin that was necessary for water-soaked lesion formation [13]. Here, we also concluded that RTX2 plays a role in cell-surface adhesion. Moreover, the ability of RTX2 to alter cell surface properties enables its interaction with diverse tissue types, such as the living cells in the apoplast and mature, non-living xylem vessel walls. Several plant-associated bacteria that have associations with the plant xylem possess one or more RTX-like proteins and future work on how RTX proteins facilitate plant xylem wall attachment and systemic colonization will provide valuable insights into the mechanisms that enable a vascular lifestyle during plant host-microbe interactions.

## Electronic supplementary material

Below is the link to the electronic supplementary material.

Supplementary Material 1

## Data Availability

All data and bacterial strains will be made available upon publication.

## Abbreviations

Bap: Biofilm-Associated Proteins
RTX: Repeat in toxin
EPS: Exopolysaccharide
LBG: Luria Bertani amended with glucose
TBSH: Tris-buffered saline, pH 7.4 with 0.1% Tween 20
ODI: Optical density initial
ODF: Optical density final
Nal: Nalidixic acid
PBS: Phosphate buffered saline
SEM: Scanning electron microscopy
RT: Reverse transcriptase
BATH: Bacterial attachment to hydrocarbons
Rcs: Regulator of capsular synthesis
