# Supplementary material for: A membrane localized RTX-like protein mediates physiochemical properties of the Pantoea stewartii subsp. stewartii cell envelope that impact surface adhesion, cell surface hydrophobicity and plant colonization
Source: BMC Microbiol. 2024 Sep 28;24:369. doi: 10.1186/s12866-024-03516-w (PMC11438254; doi:10.1186/s12866-024-03516-w)
Supplement: Supplementary file 1 — Supplementary Material 1 [file 12866_2024_3516_MOESM1_ESM.docx]

**SUPPLEMENTAL MATERIAL**


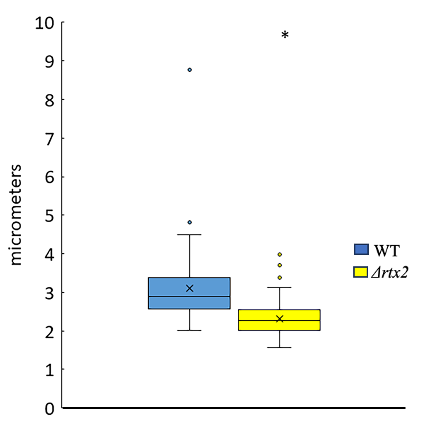


**Figure S1. Deletion of *rtx2* decreases length of the bacterial cell.** The length of individual wild type and *Δrtx2* strains containing the plasmid pHC60 (constitutively expresses GFP) were measured by a Confocal Inverted Zeiss 880 Airyscan UV PALM and Imaris x64® software (version 9.1.2; Mfg.: Bitplane). * indicate treatments that are statistically different at p ≤ 0.05 by t-test (n=90). Results are based on measurements of 30 separate cells and the experiment was repeated 3 times. The top and bottom whiskers of the box plot represent the highest and lowest values (excluding outliers), respectively. The average sample value is denoted as an X. The line between the top and bottom of the box represents the median value. The top of the box is the 3^rd^ or upper quartile (25% of observations are greater than this value). The bottom of the box denotes the 1^st^ or lower quartile (25% of observations are lower than this value).


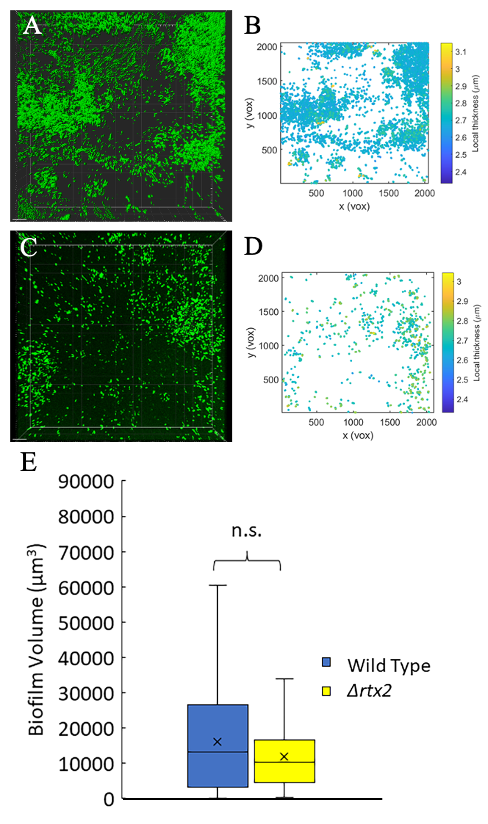


**Figure S2. Deletion of *rtx2* in a wild type *Pnss* genetic background did not impact biofilm volume.** Biofilm volume was not reduced *in* *vitro* when the *Δrtx2* deletion mutation was introduced into the wild type *Pnss* genetic background. Specifically, A and B) wild type *Pnss* biofilms had similar overall 3-dimensional volume as the C and D) *Δrtx2* strain.E) Results are based on 11 separate measurements within the biofilm of each strain and the experiment was repeated 5 times. Statistical analysis was performed via a Mann-Whitney Test (p = 0.7094; n=55). n.s=not significant. The top and bottom whiskers of the box plot represent the highest and lowest values (excluding outliers), respectively. The average sample value is denoted as an X. The line between the top and bottom of the box represents the median value. The top of the box is the 3^rd^ or upper quartile (25% of observations are greater than this value). The bottom of the box denotes the 1^st^ or lower quartile (25% of observations are lower than this value).

**
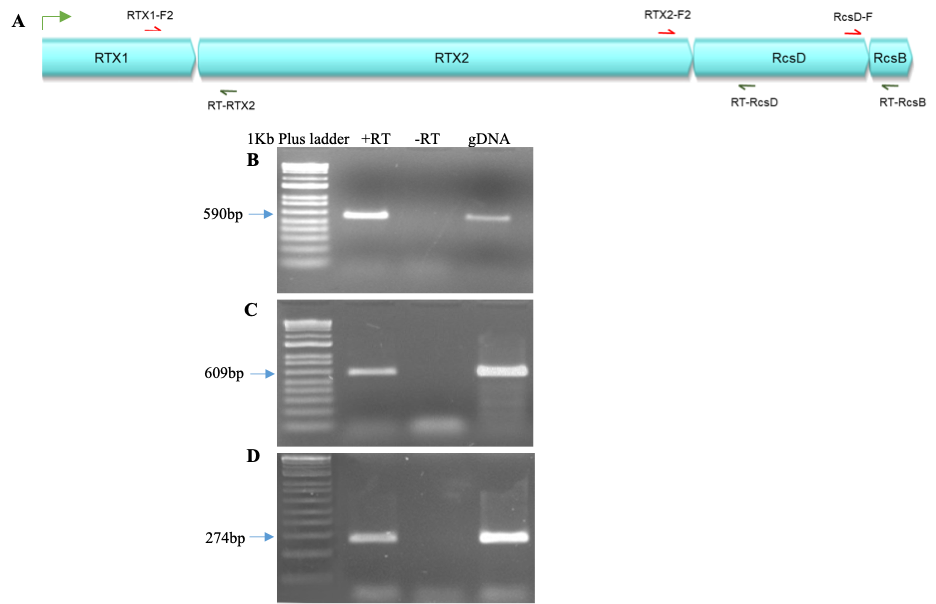
**

**Figure S3. *rtx1/rtx2/rcsD/rcsB* are co-transcribed. A**) Schematic representation of the *Pantoea stewartii* *rcsD* and *rcsB* loci with upstream *rtx1* and *rtx2* open reading frames. Small arrows indicates forward (red) and reverse (green) primer annealing sites. B-D ) Semi-quantitative RT-PCR analysis of *P. stewartiii* cDNA (+RT) ; B) minus reverse transcriptase control (-RT) and genomic DNA (gDNA). *rtx1-rtx2* co-transcription (primers: RTX1-F2 and RT-RTX2), amplicon size 590bp; C) *rtx2-rcsD* co-transcription (primers: Rtx2-F2 and RT-RcsD), amplicon size 609bp; D) *rcsD-rcsB* co-transcription (primers: RcsD-F and RT-RcsB), amplicon size 274bp. Green arrow indicates the native promoter of the operon.

**Original blot and gel images**


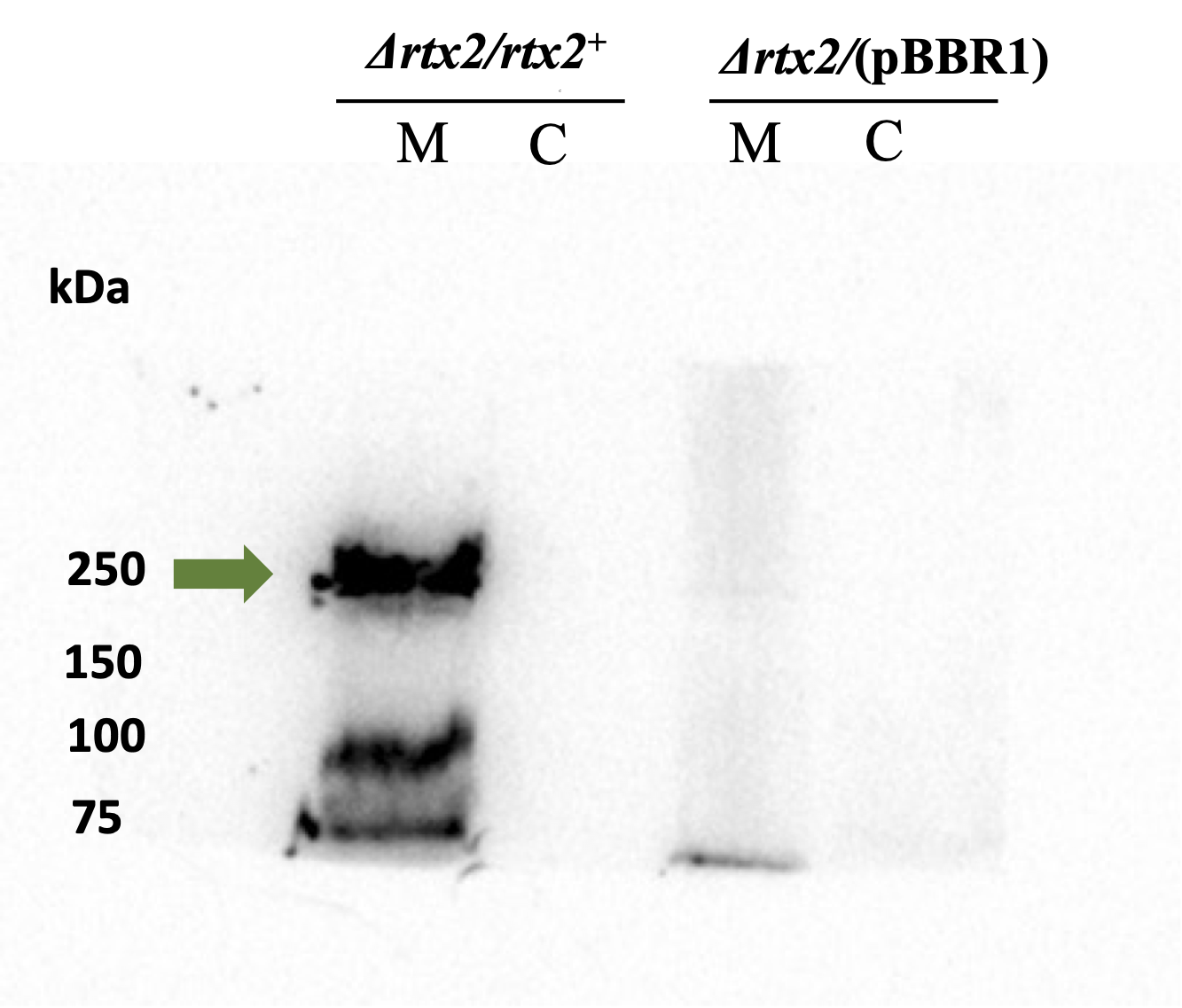


**Figure S4. Original blot for Figure 2.** RTX2 localizes to the membrane fraction of the cell**.** Rabbit polyclonal antibody raised against a peptide of the RTX2 protein (Genscript, Piscataway, NJ) detected a protein of approximately 250 kDa (RTX2= 249.8 kDa) in the membrane fraction (indicated with a green arrow) of *Pnss (∆rtx2/rtx2^+^*). This band was not detected in the cytoplasmic fraction of this strain, or in the membrane or cytoplasmic fractions of the corresponding *Δrtx2* mutant *(∆rtx2* w/ pBBR1 (empty pBBR1-MCS4 vector)). M=membrane and C=cytoplasmic.


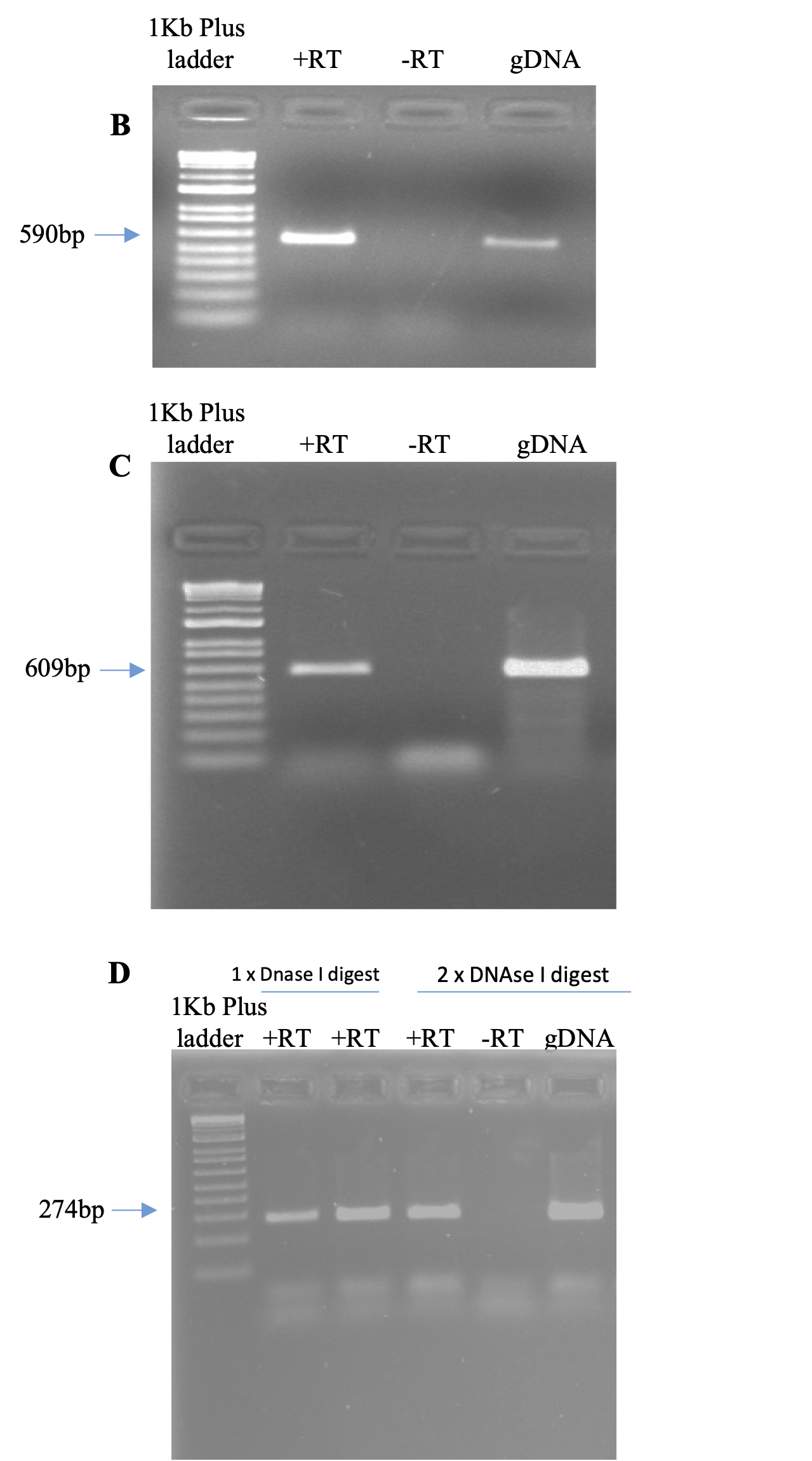


**Figure S5. Original, uncropped gels for Figure S3.** Semi-quantitative RT-PCR analysis of *P. stewartii*i cDNA (+RT) ; minus reverse transcriptase control (-RT) and genomic DNA (gDNA) RTX1-RTX2 co-transcription (primers: RTX1-F2 and RT-RTX2), amplicon size 590bp (**B**); RTX2-RcsD co-transcription (primers: Rtx2-F2 and RT-RcsD), amplicon size 609bp (**C**) RNA for both **B** and **C** were 2X DNAse treated; RcsD-RcsB co-transcription (primers: RcsD-F and RT-RcsB), amplicon size 274bp (**D**)
